# Sup35 methionine oxidation is a trigger for *de novo* [*PSI*^+^] prion formation

**DOI:** 10.1080/19336896.2015.1065372

**Published:** 2015-08-12

**Authors:** Chris M Grant

**Affiliations:** Faculty of Life Sciences; University of Manchester; Manchester, UK

**Keywords:** antioxidants, methionine oxidation, oxidative stress, prions, [*PSI*^+^], yeast

## Abstract

**ABSTRACT. **The molecular basis by which fungal and mammalian prions arise spontaneously is poorly understood. A number of different environmental stress conditions are known to increase the frequency of yeast [*PSI*^+^] prion formation in agreement with the idea that conditions which cause protein misfolding may promote the conversion of normally soluble proteins to their amyloid forms. A recent study from our laboratory has shown that the *de novo* formation of the [*PSI*^+^] prion is significantly increased in yeast mutants lacking key antioxidants suggesting that endogenous reactive oxygen species are sufficient to promote prion formation. Our findings strongly implicate oxidative damage of Sup35 as an important trigger for the formation of the heritable [*PSI*^+^] prion in yeast. This review discusses the mechanisms by which the direct oxidation of Sup35 might lead to structural transitions favoring conversion to the transmissible amyloid-like form. This is analogous to various environmental factors which have been proposed to trigger misfolding of the mammalian prion protein (PrP^C^) into the aggregated scrapie form (PrP^Sc^).

## *De novo* Formation of Yeast and Mammalian Prions

Most cases of prion diseases are sporadic, that is they form spontaneously without any underlying genetic change. The emergence of disease correlates with a novel conformational form (PrP^Sc^) of the cellular PrP^C^ protein.^1^ This prion form replicates through a cycle of seeded polymerisation and fragmentation and it is assumed that genetic or environmental factors trigger the initial conformational change in the absence of any pre-existing PrP^Sc^ ‘seeds’. The mechanism(s) underlying this switch from a normally soluble protein to a misfolded, infectious form are poorly understood. It is important that these mechanism(s) are established if we are to develop effective preventative measures for human and animal amyloidoses.

The initial alternative conformational state may be instigated by spontaneous misfolding events, triggered by mutation, mistranslation, environmental stresses and/or by disruption of the chaperone network.^2,3^ This is a relatively rare event and for example the *de novo* appearance of yeast [*PSI*^+^] prion formation has been estimated to occur at frequencies ranging between ∼10^−5^–10^−7^.^3–5^ This frequency can be elevated 1000-fold by overexpression of Sup35, presumably due to the excess Sup35 increasing the possibility for prion seed formation.^6–8^ Additionally, alterations in the protein homeostasis network including mutations in chaperones and the ubiquitin proteasome system can increase the spontaneous rate of [*PSI*^+^] prion formation.^9^ This suggests that spontaneous prion formation might arise as a consequence of random protein misfolding events which are normally dealt with by the cellular protein homeostasis systems. Conditions which cause the formation of misfolded or partially folded proteins may therefore promote the conversion of soluble proteins to their amyloid forms and several different environmental stress conditions, including oxidative stress, have been shown to increase the frequency of [*PSI*^+^] prion formation.^10^ Further support for oxidative stress conditions playing a role in *de novo* prion formation has come from the finding that [*PSI*^+^] prion formation is significantly increased in yeast mutants lacking the peroxiredoxins, Tsa1 and Tsa2.^11,12^ Peroxiredoxins are major cellular antioxidants and we have recently extended this analysis to show that the frequency of [*PSI*^+^] prion formation is commonly elevated in a range of antioxidant mutants, as well as in response to oxidant exposure.^13^ These findings strongly implicate oxidative damage of Sup35 as an important trigger for the formation of the heritable [*PSI*^+^] prion in yeast.

## Oxidative Stress and [*PSI*^+^] Prion Formation

Oxidative stress is a ubiquitous stress which occurs during the course of normal aerobic metabolism or following exposure to radical-generating compounds.^14^ Reactive oxygen species (ROS) cause wide-ranging damage to macromolecules and an oxidative stress is said to occur when ROS overwhelm the cellular antioxidant defense systems. Oxidative stress is frequently implicated in disease and pathological conditions, although it is not always easy to separate cause from effect. ROS are also a well-established trigger of protein misfolding and damage proteins via oxidative modification of several different amino acid residues.^15^

Increasing evidence suggests that protein oxidative damage can trigger misfolding events resulting in prion formation. Oxidative stress induced by exposure to hydrogen peroxide was found to significantly increase [*PSI*^+^] prion induction in a screen which used a Sup35 variant with increased [*PSI*^+^] formation rates relative to wild-type Sup35.^10^ This study found that exposure to higher; more lethal concentrations of hydrogen peroxide (10 mM), were required for maximal [*PSI*^+^] induction. We have found that growth in the presence of lower concentrations of hydrogen peroxide (100 μM), which only modestly affect wild-type yeast growth, induce *de novo* formation of [*PSI*^+^] from wild-type Sup35.^13^ Stronger induction of [*PSI*^+^] prion formation is also observed in response to oxidative stress caused by the superoxide anion.^13^ However, the superoxide anion can be dismutated to hydrogen peroxide by the activity of superoxide dismutases and both hydrogen peroxide and the superoxide anion are thought to oxidize amino acid residues through the generation of the highly reactive hydroxyl radical.^16^ More work will be required to determine the sources and types of ROS which promote protein damage leading to prion formation, which is important since no single oxidant is typical of ‘oxidative stress’.^17^

An unbiased genome-wide screen for factors which increase [*PSI*^+^] induction did not identify any antioxidants.^10^ However, this screen differentiated [*PSI*^+^] formation from nuclear gene mutations by their irreversible elimination in guanidine hydrochloride (GdnHCl). Reversible curability is a well-established genetic criterion for yeast prions^6^ and is commonly tested using GdnHCl which blocks the propagation of prions by inhibiting the key ATPase activity of Hsp104, a molecular chaperone that is absolutely required for yeast prion propagation.^18,19^ The gene deletions identified in this screen were all curable at levels above 95% suggesting that they did not cause any increase in nuclear mutations. All of the antioxidant mutants which we have shown increase the frequency of spontaneous [*PSI*^+^] formation also exhibit significantly increased rates of nuclear mutations.^13^ ROS are a well-known cause of DNA damage resulting in increased rates of mutagenesis and genomic instability. For example, loss of *TSA1* results in an increased rate of spontaneous mutations which arise as a result of endogenous ROS production.^20^ However, there does not appear to be any correlation between the frequency of nuclear mutations and the frequency of [*PSI*^+^] prion formation in antioxidant mutants.^13^

## Methionine Oxidation Triggers [*PSI*^+^] Prion Formation

**Figure 1.**
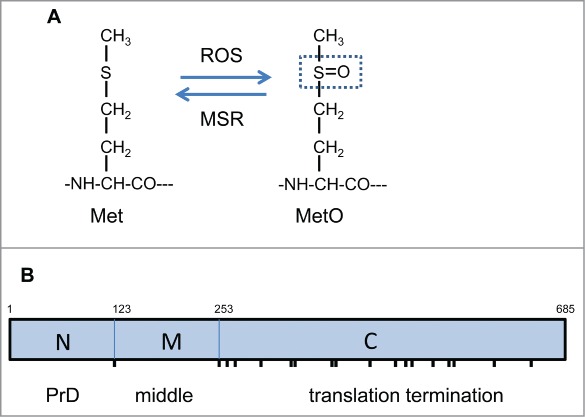
Methionine oxidation and Sup35 functional domains. (**A**) Oxidation converts the moderately hydrophobic thioester side chain of methionine into the hydrophilic sulphoxide form (MetO). Methionine oxidation can be reversed by the activity of MSR enzymes which catalyze the thiol-dependent reduction of MetO. (**B**) The Sup35 protein can be divided into 3 distinct regions: an N-terminal prion forming domain (PrD), a highly charged middle region (M) and a C-terminal domain which functions in translation termination (C). The N-terminal domain lies between Met1 and Met123, the M-domain between Met123 and Met253 and the C-terminal domain from Met253 until the C-terminus of the protein. Sup35 contains a total of 19 Met residues (including its N-terminal Met residue) which are marked as vertical lines: the N and M regions contain no Met residues, the M region is flanked by Met residues and the C-terminal domain contains 16 Met residues.

All amino acids are potential targets for oxidation, but methionine residues are particularly sensitive forming a racemic mixture of methionine-*S*-sulphoxide and methionine-*R*-sulphoxide in cells.^21^ Oxidation converts the moderately hydrophobic thioester side chain of methionine into the hydrophilic sulphoxide form (MetO) which can significantly influence protein folding and structure (**Fig. 1A**). Methionine oxidation can be reversed by the activity of methionine sulfoxide reductases (MSR) and hence methionine oxidation has been proposed to play an antioxidant role in scavenging ROS.^22^ A number of proteins have been identified which form MetO in response to oxidative stress, including many examples where oxidation is thought to play a regulatory role modulating protein activity.^23^ Thus, MetO formation is increasingly thought of as a reversible post translational modification, as well as a marker of protein oxidative damage.

We have used an anti-MetO antibody to detect methionine oxidation of Sup35. Oxidative stress conditions induced by exposure to hydrogen peroxide or the superoxide anion significantly increased MetO levels and increased the frequency of [*PSI*^+^] prion formation in a [*PIN*^+^][*psi*^−^] strain.^13^ Similar increases in MetO levels were detected in antioxidant mutants, including mutants deficient in superoxide dismutases, catalases and peroxiredoxins, which all display increased [*PSI*^+^] prion formation. Increased MetO levels were detected in Sup35 from antioxidant mutants grown under normal non-stress conditions indicating that endogenous ROS levels are sufficient to promote Sup35 oxidation. Increased methionine oxidation is commonly detected in a number of disease states as well as during the aging process. For example, MetO antibodies have been used to detect increased methionine oxidation in aged mouse samples and samples from patients with Alzheimer's disease.^24^ The MetO content of proteins is also known to increase with age in various model systems.^25^ Thus, increased MetO content appears to be a common feature of oxidative stress conditions during various pathological conditions.

It is not know which methionine residues in Sup35 are important for [*PSI*^+^] prion formation. Sup35 has 3 functional regions: an N-terminal region (residues 1–123) which is essential for prion formation, a highly charged middle (M) region (residues 124–253) and a C-terminal region (residues 254–685) which is required for translation termination activity.^26^ Sup35 contains a total of 19 Met residues including its N-terminal Met residue (**Fig. 1B**). The N and M regions contain no Met residues, but the M region is flanked by Met residues. Although the C domain of Sup35 does not form the [*PSI*^+^] prion in the absence of the NM region, oxidation of Met residues in the C domain might be expected to affect the overall protein structure/folding of Sup35 which in turn might promote the formation of misfolded structures that have a higher propensity to switch to the prion conformation. Alternatively Met mutations may affect specific Sup35-chaperone interactions. For example, the M region interacts with Hsp104 and is required for prion curing but not propagation.^27^ Several chaperones are known to modulate *de novo [PSI*^+^] prion formation, although the molecular details are not well defined.^9^ Hence, methionine oxidation and any resulting alterations in Sup35 secondary structure which alter protein-protein interactions may affect prion formation.

## How does Oxidative Protein Damage Promote [*PSI*^+^] Formation?

The elevated frequency of [*PSI*^+^] formation observed in response to Sup35 overexpression requires the presence of another prion, the best studied example of which is the [*PIN*^+^] prion form of Rnq1.^28^ [*PIN*^+^] is still required for the elevated frequency of [*PSI*^+^] prion formation observed in *tsa1 tsa2* mutants, indicating that oxidative stress does not overcome the requirement of cells being [*PIN*^+^] to form the [*PSI*^+^] prion.^12^ [*PIN*^+^] prion aggregates are thought to provide imperfect templates which seed the polymerization of Sup35, facilitating the formation of its transmissible prion form.^29^ Recent studies have provided a better understanding of the pathway that leads to prion formation. Misfolded Sup35 molecules are targeted to the IPOD (Insoluble Protein Deposit), a component of the protein quality control machinery in eukaryotic cells that is located adjacent to the vacuole. The IPOD is a site of accumulation of both prion proteins, as well as oxidatively damaged proteins.^30,31^ This is believed to facilitate the nucleation of prion protein polymerization in a [*PIN*^+^]-dependent manner.^32^ The transmissible form of the prion is then formed via a 2 stage process which initially involves the formation of non-transmissible extended polymers of the prion protein followed by their fragmentation into shorter transmissible forms that are thought to define the propagon.^30^

**Figure 2.**
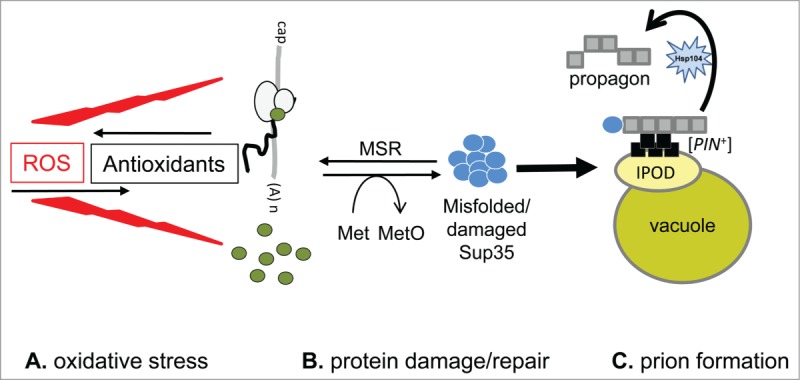
Model depicting the role of antioxidants in protecting Sup35 against protein oxidation and *de novo* [*PSI*^+^] prion formation. Oxidative stress occurs when ROS overwhelm the cellular antioxidant defense systems. Such stress can damage all macromolecules in cells including amino acid residues in proteins. Methionine residues are particularly sensitive forming MetO. It remains to be established whether methionine oxidation occurs on the nascent Sup35 polypeptide chain or in pre-existing Sup35 proteins. Sup35 is shown as green dots on translating ribosomes or free in cells. Antioxidants therefore provide the first line of defense against Sup35 oxidation and misfolding. When MetO formation does occur on Sup35, it causes misfolding and aggregation which may underlie the switch from a soluble to an aggregated form of Sup35. Methionine sulphoxide reductases (MSR) provide a second line of defense by converting MetO back to methionine. Once oxidized, Sup35 misfolds and the resulting aggregates can be targeted to the IPOD where [*PIN*^+^] aggregates cross-seed *de novo* formation of the [*PSI*^+^] prion. Soluble Sup35 is converted into the [*PSI*^+^] state which interacts with additional monomeric forms of Sup35. The growing amyloid fibrils are fragmented and propagated by the disaggregase activity of the Hsp104 chaperone. The resulting propagons are inherited in daughter cells via cytoplasmic transfer which ensures continued prion propagation.

Our data indicate that while methionine oxidation can promote prion formation, it can be reversed by the activity of MSR enzymes preventing the switch to the heritable prion form. Yeast contains 3 MSR enzymes (fRMsr/MsrA/MsrB).^33^ Overexpression of MsrA protects Sup35 against methionine oxidation and also prevents [*PSI*^+^] prion formation, confirming that Sup35 oxidation can cause the switch from a soluble to an aggregated prion-form of Sup35 in antioxidant mutants.^12,13^ Taking these observations together we propose a model for ROS-induced [*PSI*^+^] prion formation as shown in **Figure 2**. Oxidation of Sup35 promotes misfolding and the resulting misfolded/damaged forms of Sup35 are targeted to the IPOD where [*PIN*^+^] aggregates promote *de novo* formation of the [*PSI*^+^] prion. Sup35 oxidation may cause misfolding by altering the conformation of its polypeptide backbone and decreasing thermal stability, or alternatively, it may disrupt normal Sup35-chaperone interactions. It is unknown whether Sup35 oxidation occurs on the nascent polypeptide chain or in pre-existing Sup35 molecules (**Fig. 2**). Interestingly however, Tsa1 and Tsa2 are both ribosome-associated antioxidants where they may localize to protect nascent proteins as well as the protein biosynthetic machinery against oxidation.^11,34^

Under normal non-stress growth conditions, MetO formation is not detected in Sup35 suggesting that cellular antioxidants are sufficient to protect against endogenous ROS exposure and MSR enzymes function to reduce any MetO that might be formed. The addition of an exogenous oxidant, which can overwhelm the cellular antioxidant defense systems, results in Sup35 oxidation and misfolding, prior to targeting to the IPOD where it is converted to the heritable [*PSI*^+^] prion form. Methionine oxidation does not appear to account for the formation of all yeast prions since we were unable to detect methionine oxidation of Rnq1 in a *tsa1 tsa2* mutant.^12^ However, the frequency of [*PIN*^+^] formation is elevated in *tsa1 tsa2* and *sod1* mutants suggesting that prion formation may be a common phenomenon in antioxidant mutants.^12,13^ The spontaneous frequency of [*PIN*^+^] prion formation is significantly higher than [*PSI*^+^] prion formation^12^ and hence it may be difficult to detect methionine oxidation once it has formed the [*PIN*^+^] prion form. Alternatively, other forms of protein oxidation may underlie the switch from soluble Rnq1 to its amyloid form.

## Conflicting Evidence Linking Methionine Oxidation with Mammalian Prion Formation

Conflicting data has been reported regarding the potential role of methionine oxidation in the aggregation and pathogenicity of mammalian prions. Mass spectrometry was originally used to detect MetO in PrP^Sc^ purified from brain samples; although it was unclear whether oxidation actually occurred in the brain or during the protein purification and analysis protocol.^35^ Exposing purified PrP^C^ to an oxidant has been shown to result in methionine oxidation,^36^ although other *in vitro* studies suggested that methionine oxidation of recombinant hamster and mouse PrP^C^ interferes with its conversion into amyloid fibrils.^37^ Further *in vivo* studies showed that MetO (particularly Met213 in humans) could be detected in PrP^Sc^ isolated from brain samples, but not in PrP^C^, leading to the suggestion that MetO is a specific marker for PrP^Sc^.^38^ Conversely, sensitive mass spectrometry based comparison of Met213 oxidation in PrP^C^ and PrP^SC^ isolated from a hamster-adapted scrapie model found comparable low levels of methionine oxidation suggesting that MetO213 should not be regarded as a prion-specific covalent signature.^39^ Assuming that methionine oxidation does occur on the PrP protein *in vivo*, it raise the question as to whether methionine oxidation underlies the structural switch that converts PrP^C^ to PrP^Sc^, or is simply a biomarker for PrP^SC^ formation. Specificity for PrP^Sc^ might arise if methionine oxidation requires partial denaturation of PrP^C^ before methionine residues can become oxidized, or else they may be oxidized only after PrP^SC^ forms.

None of the existing *in vivo* studies are able address the importance of methionine oxidation in sporadic PrP^SC^ formation. *In vitro* studies have attempted to examine the importance of methionine oxidation as a destabilizing event that triggers PrP^C^ misfolding leading to spontaneous prion formation. For example, methionine analogs have been used to correlate the degree of PrP^C^ methionine oxidation with the structural transitions that underlie its conversion to the infectious PrP^SC^ form.^40^ More recently, methionine oxidation within the core of PrP^C^ has been shown to reduce its thermal stability, promoting generation of a molten globule fold which is thought to be a key step in the misfolding pathway.^41^ Similarly, molecular dynamic simulations have suggested that oxidation of Met213 triggers destabilization of PrP^C^ which can trigger its conversion to the pathogenic PrP^SC^ form.^42,43^ Pseudosulfoxidation mutants have been used to mimic methionine oxidation, confirming that oxidation of surface exposed methionine residues perturbs the PrP^C^ structure resulting in destabilization and misfolding.^44^ Interestingly, the D178N variant of PrP^C^ was recently found to be more susceptible to methionine oxidation.^45^ This PrP mutant has been linked with the inherited human prion disease fatal famililial insomnia (FFI) and methionine oxidation of D178N PrP^C^ decreased its structural stability, enhanced its aggregation and increased neurotoxicity. This suggests that methionine oxidation can have synergistic effects with other pathogenic mutations in PrP^C^ which cause prion diseases.

Further work will be required to establish a causal role for methionine oxidation in PrP misfolding and the formation of toxic oligomers and amyloid plaques. This may occur via PrP^C^ destabilization or PrP^SC^ stabilization such as inhibiting clearance of misfolded forms and favoring misfolding pathways which result in amyloid formation. This will however, be experimentally difficult without the tractability of the yeast model system.

## Disclosure of Potential Conflicts of Interest

No potential conflicts of interest were disclosed.
